# College and Hospital Notes

**Published:** 1903-12

**Authors:** 


### COLLEGE AND HOSPITAL NOTES.

The D. W. Harrington Lectureship, of the University of Buffalo, founded by Dr. D. W. Harrington of this city, has been established and the medical faculty has chosen Dr. Samuel J. Meltzer, of New York, to deliver these lectures for 1903. The subject selected by Dr. Meltzer is, Edema, a consideration of the physiological and pathological factors concerned in its formation. The lectures will be delivered in the Medical College, November 30 and December 1, 2 and 3, at 5 p. m. The medical profession is cordially invited to attend. These are the first lectures given on this foundation. Lectures will be given as often as the income from the fund will warrant.

The amalgamation of the medical faculties of Toronto and Trinity Universities has become an accomplished fact, and the opening of the new buildings of the University of Toronto was celebrated in appropriate fashion October 1, 1903. Representatives were present from sister universities in the United States and Canada, and a dedicatory address was delivered by Professor Charles H. Sherrington, M. D., Hall professor of physiology in the University of Liverpool. Other addresses were delivered by Welch, of Johns Hopkins; Bowditch, of Harvard; Chittenden, of Yale; Roddick, of McGill; Abbott, of Pennsylvania; McMurrich, of Michigan; Barker, of Chicago; Osler, of Johns Hopkins, and Park, of Buffalo. The Canadian *Practitioner* for November, devotes its entire space to a detailed account of the proceedings, publishing all the addresses and lectures in full, and giving illustrations of the buildings. It is a creditable exhibition of journalistic enterprise.

The Memorial Hospital, Richmond, Va., was opened for patients August 1, 1903. The building is a stately modern structure, built on classic lines and is one of the most complete hospitals in the south. A beautiful brochure has just been issued by the hospital authorities, which is a model of exquisite taste, descriptive of the hospital and its purposes, illustrated with numerous engravings, showing the exterior and interior of the building. Dr. George Ben Johnston supervised the plans and the construction of the building, and deserves great credit for the admirable result.

---

The Western Surgical and Gynecological Association will hold its thirteenth annual meeting at Denver, Colo., December 28 and 29, 1903, under the presidency of Alexander Hugh Ferguson, Chicago. The preliminary program has been issued, additions to which may be made until December 10, by addressing the secretary, Dr. George H. Simmons, 103 Dearborn avenue, Chicago.

---

The New York State Association of Railway Surgeons held its thirteenth annual meeting at the New York Academy of Medicine, November 12, 1903, under the presidency of Dr. Henry Flood, of Elmira. Drs. G. R. Trowbridge, F. J. Carr and A. J. Gumaer, of Buffalo, attended the meeting and invited the association to come to this city next year, but New York has been selected as the permanent meeting place. Dr. Trowbridge read a paper entitled, Conservatism in railway surgery. A change of name was made to the New York and New England Association of Railway Surgeons.

